# Long-term Body Weight Gain After Maturity is Associated With the Incidence of Chronic Kidney Disease (CKD), Independent of Current Body Weight, in Middle-aged and Older Men

**DOI:** 10.2188/jea.JE20170304

**Published:** 2019-06-05

**Authors:** Ryoma Michishita, Takuro Matsuda, Shotaro Kawakami, Satoshi Tanaka, Akira Kiyonaga, Hiroaki Tanaka, Natsumi Morito, Yasuki Higaki

**Affiliations:** 1 Department of Health Development, Institute of Industrial Ecological Sciences, University of Occupational and Environmental Health, Kitakyushu, Japan; 2 Fukuoka University Institute for Physical Activity, Fukuoka, Japan; 3 Department of Rehabilitation, Fukuoka University Hospital, Fukuoka, Japan; 4 Laboratory of Exercise Physiology, Faculty of Health and Sports Science, Fukuoka University, Fukuoka, Japan; 5 Fukuoka University Health Care Center, Fukuoka, Japan; 6 Department of Cardiology, Fukuoka University School of Medicine, Fukuoka, Japan

**Keywords:** incidence of CKD, long-term body weight gain, current body weight, health checkup

## Abstract

**Background:**

This study investigated the relationship between long-term body weight gain after maturity and the incidence of chronic kidney disease (CKD).

**Methods:**

The participants were 303 men without a history of cardiovascular and cerebrovascular diseases, kidney dysfunction, or dialysis treatment. Their body weight gain after maturity was examined using a standardized self-administered questionnaire. The participants were divided into two groups based on the presence/absence of a body weight gain of ≥10 kg since 20 years of age.

**Results:**

After a 6-year follow-up, the cumulative incidence of CKD was significantly higher in participants with a body weight gain of ≥10 kg than in participants without body weight a body weight gain of ≥10 kg since 20 years of age (log-rank test: *P* = 0.041). After adjusting for the age, body mass index, estimated glomerular filtration rate levels, smoking and drinking habits, and the presence of hypertension, dyslipidemia, and hyperglycemia at baseline, the normal body weight participants with a body weight gain of ≥10 kg since 20 years of age was significantly related to the incidence of CKD (hazard ratio 2.47; 95% confidence of interval, 1.02–6.01, *P* = 0.045).

**Conclusions:**

These results suggest that long-term body weight gain after maturity in normal body weight participants may be associated with the incidence of CKD, independent of current body weight.

## INTRODUCTION

It is well known that the chronic kidney disease (CKD) is a risk factor for the progression of end-stage renal disease (ESRD) and cardiovascular morbidity and mortality.^1^^,^^2^ At present, the large number of ESRD patients is thought to be associated with the increasing number of patients with CKD. Non-communicable diseases, such as type 2 diabetes mellitus (DM), hypertension, dyslipidemia, and metabolic syndrome, are known to be common disorders.^3^ In addition, the risk factors for CKD progression are thought to be aging, obesity, hypertension, type 2 DM, and MetS.^4^^–^^8^ In our previous study,^9^ we found that hypertension and hyperglycemia alone and in combination were associated with the progression of CKD. Recently, the long-term body weight gain after maturity has been related to the progression of type 2 DM, metabolic syndrome, cardiovascular disease (CVD), and cardiovascular mortality, independent of current body weight.^10^^–^^15^ However, at present, the influence of the long-term body weight gain after maturity on the development of CKD has not been clarified, despite the fact that the long-term body weight gain after maturity has been associated with the progression of type 2 DM, metabolic syndrome, CVD, and cardiovascular mortality.^10^^–^^15^

We hypothesized that the long-term body weight gain after maturity might predict the incidence of CKD. In Japan, the number of obese participants has been continuously increasing.^16^ The aim of body weight control is not only to improve obesity but also to inhibit the progression of ESRD and CVD. Therefore, clarifying the influence of the long-term body weight gain after maturity on the incidence of CKD may highlight the importance of CKD prevention. This retrospective study examined the long-term body weight gain after maturity on the incidence of CKD.

## METHODS

### Participants

**Figure 1.**
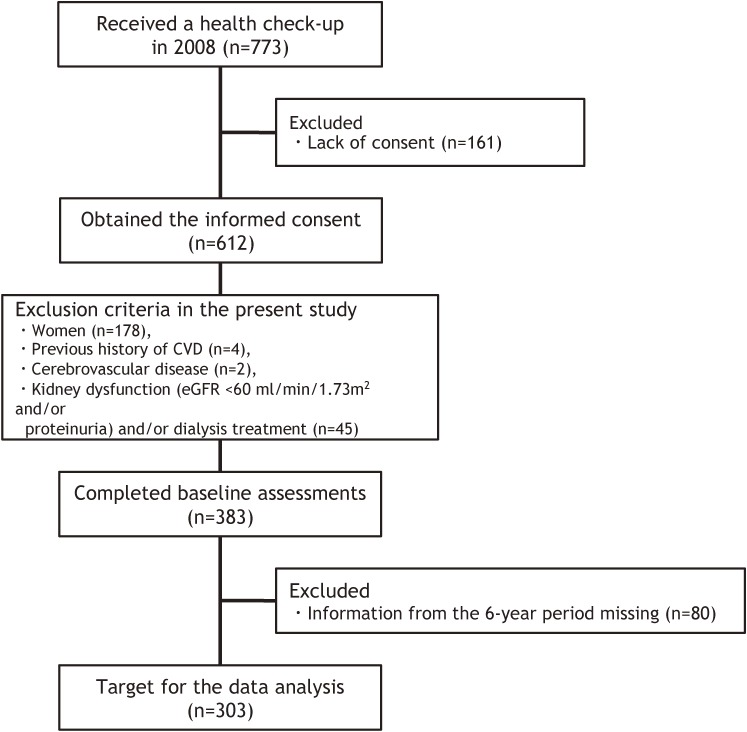
A flow-chart of the participants associated with the present study.

A total of 773 middle-aged and older participants underwent their regular health checkup in 2008. The protocol for the present study has been described in previous studies.^9^^,^^17^^–^^19^ Figure 1 shows a flow chart of the participants who were associated with this study. Among the 612 participants who provided their informed consent, women (*n* = 178) and participants with a past history of CVD (*n* = 4) and cerebrovascular disease (*n* = 2) were excluded from the analysis to eliminate the influence of sex difference and complications, because it is well known that subjects with CVD and cerebrovascular disease have many complications. Participants with the kidney dysfunction (glomerular filtration rate estimated by the Japanese glomerular filtration rate inference formula [eGFR] <60 mL/min/1.73 m^2^, or proteinuria, or both)^20^ and/or past history of dialysis treatment (*n* = 45) were also removed from the analysis. A total of 303 men (mean age, 52.2 [standard deviation {SD}, 6.7] years; mean body mass index [BMI], 23.4 [SD, 2.8] kg/m^2^; mean serum creatinine (Cr), 0.84 [SD, 0.09] mg/dL, and mean eGFR, 77.0 [SD, 10.3] mL/min/1.73 m^2^) without data loss over the previous 6 years were qualified for inclusion in this study. In this study, participants taking medications were included (anti-hypertensive drugs users, *n* = 43; anti-hyperlipidemic agents users, *n* = 25; hypoglycemic agents users, *n* = 7).

All of the participants gave their informed consent to participate after agreeing with the aim, design, and significance of this research. The study conformed to the Declaration of Helsinki guidelines and was recognized by the Ethics Committee of our University (No. 11-08-01).

### Classification of CKD

The methods of blood sampling and anthropometry measurement have been shown in our previous studies.^9^^,^^17^^–^^19^ Blood samples were collected early in the morning, after at least 12 h of fasting, from an antecubital vein using venipuncture. The blood samples were analyzed by Special Reference Laboratories (SRL Inc., Tokyo, Japan).

The grade of CKD was classified according to the eGFR and the presence of proteinuria. The eGFR was calculated using the Japanese GFR inference formula as follows: eGFR (mL/min/1.73 m^2^) = 194 × serum Cr (mg/dL)^−1.094^ × age (years)^−0.287^.^21^ A urinalysis was carried out using a dipstick, and results of urine test were classified as (−), (±), (1+), (2+) and (3+).^22^ The CKD was classified based on definition of the Japanese Society of Nephrology as follows: eGFR <60 mL/min/1.73 m^2^, or positive proteinuria (more than 1+), or both.^20^ The classification of the CKD grade^20^ of the participants at baseline was as follows: G1 (eGFR ≥90 mL/min/1.73 m^2^), *n* = 29 (9.6%); and G2 (eGFR 60–89 mL/min/1.73 m^2^), *n* = 274 (90.4%).

Overweight was classified according to the guideline of the Japan Society for the Study of Obesity (BMI ≥25.0 kg/m^2^).^23^ Metabolic syndrome was defined based on the metabolic syndrome diagnostic criteria of the Japanese Society for Internal Medicine.^24^^,^^25^

### Assessment of long-term body weight gain after maturity

The participants’ lifestyle habits were determined according to the standardized self-administered questionnaire of the National Health Promotion Program.^26^^,^^27^ Previously, we have shown that the combination of healthy lifestyle habits regarding regular exercise and diet are associated with the progression of CKD.^17^^–^^19^ The long-term body weight gain after maturity was assessed according to their responses to the following questionnaire item: a body weight gain of ≥10 kg since 20 years of age (yes or no).

### Statistical analyses

The participants received their periodic health checkup once a year. The endpoint of the present study was the onset year of CKD, and a blood sampling and evaluation of lifestyle habits were administered at baseline (2008) to endpoint year (2014, for a maximum of 6 years). The StatView J-5.0 software package (SAS Institute, Cary, NC, USA) was used for all of the statistical analyses. Two-group comparisons were carried out using the Mann-Whitney’s U-test for continuous variables and the chi-squared test for categorical variables. The cumulative incidence of CKD was determined using Kaplan-Meier survival curves and the log-rank test. A Cox proportional hazards model was used to predict the incidence of CKD using the data as categorical variables. This analysis was adjusted for the following factors: age, BMI, eGFR levels, the presence of hypertension (resting systolic blood pressure [SBP] ≥140 mm Hg and/or diastolic blood pressure [DBP] ≥90 mm Hg and/or taking anti-hypertensive drugs), dyslipidemia (low-density lipoprotein cholesterol [LDL-C] ≥140 mg/dL and/or high-density lipoprotein cholesterol [HDL-C] <40 mg/dL and/or triglycerides ≥150 mg/dL and/or taking anti-hyperlipidemic agents), hyperglycemia (fasting plasma glucose ≥110 mg/dL and/or hemoglobin A_1_c [HbA_1_c; NGSP values] ≥6.5% and/or taking hypoglycemic drugs) and metabolic syndrome, and smoking and drinking habits at baseline. A probability value of <0.05 was defined to indicate statistical significance.

## RESULTS

During the follow-up, incident CKD (eGFR <60 mL/min/1.73 m^2^ and/or proteinuria) was shown in 32 participants (10.6%). The follow-up period averaged 5.7 (SD, 0.9) years. The CKD grades^20^ of the participants after follow-up were as follows: G1 (eGFR ≥90 mL/min/1.73 m^2^), *n* = 10 (3.3%); G2 (eGFR 60–89 mL/min/1.73 m^2^), *n* = 261 (86.1%); and G3a (eGFR 45–59 mL/min/1.73 m^2^), *n* = 32 (10.6%; including 2 with proteinuria). Table 1 shows the basal characteristics of participants with and without a body weight gain of ≥10 kg since 20 years of age. In the participants with a body weight gain of ≥10 kg, body weight, BMI, waist circumference, SBP, DBP, LDL-C, triglyceride, fasting plasma glucose, HbA_1_c levels, and rate of participants with hypertension, dyslipidemia, and metabolic syndrome were significantly higher and the HDL-C level significantly lower compared with the participants without a body weight gain of ≥10 kg since 20 years of age (*P* < 0.05).

**Table 1.** The basal characteristics in participants with and without a body weight gain of ≥10 kg since 20 years of age

	All (*n* = 303)	Body weight gain of ≥10 kg since 20 years of age		*P* value

		Yes (*n* = 89)	No (*n* = 214)	
eGFR, ml/min/1.73 m^2^	77.0 (10.3)	76.4 (11.5)	77.2 (9.8)	0.531
Classifications of CKD grade				
G1: eGFR ≥90 mL/min/1.73 m^2^, *n* (%)	29 (9.6)	8 (9.0)	21 (9.8)	0.824
G2: eGFR 60–89 mL/min/1.73 m^2^, *n* (%)	274 (90.4)	81 (91.0)	193 (90.2)	
Serum creatinine, mg/dL	0.84 (0.09)	0.85 (1.00)	0.84 (0.09)	0.391
Age, years	52.2 (6.7)	52.2 (5.9)	52.2 (7.0)	0.976
Body weight, kg	67.6 (9.3)	72.0 (8.2)	65.7 (9.2)	<0.0001
BMI, kg/m^2^	23.4 (2.8)	24.9 (2.3)	22.7 (2.7)	<0.0001
Waist circumference, cm	83.5 (7.6)	87.8 (5.7)	81.8 (7.6)	<0.0001
SBP, mm Hg	126.8 (15.4)	131.1 (13.6)	125.0 (15.8)	0.002
DBP, mm Hg	83.0 (10.4)	86.6 (10.0)	81.5 (10.2)	<0.0001
LDL-C, mg/dL	118.4 (25.2)	127.3 (26.6)	114.7 (23.6)	<0.0001
HDL-C, mg/dL	58.2 (13.3)	52.8 (10.9)	60.4 (13.6)	<0.0001
Triglyceride, mg/dL	115.0 (69.9)	148.9 (81.8)	100.9 (58.9)	<0.0001
Fasting plasma glucose, mg/dL	100.5 (18.1)	103.8 (22.9)	99.1 (15.6)	0.042
HbA_1_c, NGSP values; %	5.6 (0.7)	5.7 (1.0)	5.6 (0.5)	0.048
Smoking habit, yes/no; *n* (%)	63 (20.8)/240 (79.2)	22 (24.7)/67 (75.3)	41 (19.2)/173 (80.8)	0.277
Drinking habit, yes/no; *n* (%)	232 (76.6)/71 (23.4)	70 (78.7)/19 (21.3)	162 (75.7)/52 (24.3)	0.581
Hypertension, yes/no; *n* (%)	93 (30.7)/210 (69.3)	35 (39.3)/54 (60.7)	58 (27.1)/156 (72.9)	0.036
Dyslipidemia, yes/no; *n* (%)	122 (40.3)/181 (59.7)	53 (59.6)/36 (40.4)	69 (32.2)/145 (67.8)	<0.0001
Hyperglycemia, yes/no; *n* (%)	50 (16.5)/253 (83.5)	19 (21.3)/70 (78.9)	31 (14.5)/183 (85.5)	0.114
Metabolic syndrome, yes/no; *n* (%)	42 (13.9)/261 (86.1)	24 (27.0)/65 (73.0)	18 (8.4)/196 (91.6)	<0.0001
Anti-hypertensive drugs, yes/no; *n* (%)	43 (14.2)/260 (85.8)	15 (16.9)/74 (83.1)	28 (13.1)/186 (86.9)	0.392
Anti-hyperlipidemic agents, yes/no; *n* (%)	25 (8.3)/278 (91.7)	9 (10.1)/80 (89.9)	16 (7.5)/198 (92.5)	0.448
Hypoglycemic drugs, yes/no; *n* (%)	7 (2.3)/296 (97.7)	2 (2.2)/87 (97.8)	5 (2.3)/209 (97.7)	0.962

The data are presented as the mean value (standard deviation) and the number of subjects.

The classifications of CKD grade were defined according to the definition of the Japanese Society of Nephrology. CKD, chronic kidney disease; eGFR, estimated-glomerular filtration rate; BMI, body mass index; SBP, systolic blood pressure; DBP, diastolic blood pressure; LDL-C, low-density lipoprotein cholesterol; HDL-C, high-density lipoprotein cholesterol; HbA_1_c, hemoglobin A_1_c; NGSP, national glycohemoglobin standardization program.

The participants’ long-term body weight gain after maturity was determined based on their responses to the following questionnaire item: a body weight gain of ≥10 kg since 20 years of age (yes or no). Metabolic syndrome was defined according to the metabolic syndrome diagnostic criteria of the Japanese Society for Internal Medicine.^24^^,^^25^

Table 2 shows the basal characteristics of participant who did and did not progress to CKD. In the CKD group, the serum Cr level, age, SBP, DBP, fasting plasma glucose, HbA_1_c levels, and rate of subjects with a body weight gain of ≥10 kg since 20 years of age, hypertension, hyperglycemia, anti-hypertensive drug use, and anti-hyperlipidemic drug use were significantly higher and the eGFR and HDL-C levels significantly lower compared with the non-CKD group (*P* < 0.05).

**Table 2.** The basal characteristics in participants with and without the progression of CKD

	Progressed CKD (*n* = 32)	Did not progress CKD (*n* = 271)	*P* value
eGFR, mL/min/1.73 m^2^	66.8 (5.3)	78.2 (10.1)	<0.0001
Classifications of CKD grade			
G1: eGFR ≥90 ml/min/1.73 m^2^, *n* (%)	0 (0)	29 (10.7)	0.052
G2: eGFR 60–89 mL/min/1.73 m^2^, *n* (%)	32 (100)	242 (89.3)	
Serum creatinine, mg/dL	0.93 (0.06)	0.83 (0.09)	<0.0001
Age, years	54.6 (6.5)	51.9 (6.7)	0.030
Body weight, kg	67.7 (9.0)	67.5 (9.4)	0.902
BMI, kg/m^2^	23.3 (2.7)	23.4 (2.8)	0.921
Body weight gain of ≥10 kg since 20 years of age, yes/no; *n* (%)	15 (46.9)/17 (53.1)	74 (27.3)/197 (72.7)	0.022
Waist circumference, cm	84.6 (7.2)	83.4 (7.6)	0.379
SBP, mm Hg	133.7 (15.2)	126.0 (15.2)	0.007
DBP, mm Hg	86.6 (9.4)	82.6 (10.5)	0.038
LDL-C, mg/dL	119.4 (25.3)	118.3 (25.1)	0.819
HDL-C, mg/dL	53.7 (11.0)	58.7 (13.5)	0.043
Triglyceride, mg/dL	132.0 (124.0)	113.0 (60.4)	0.145
Fasting plasma glucose, mg/dL	107.1 (30.1)	99.7 (16.1)	0.030
HbA_1_c, NGSP values; %	5.9 (0.9)	5.6 (0.7)	0.031
Smoking habit, yes/no; *n* (%)	5 (15.6)/27 (84.4)	58 (21.4)/213 (78.6)	0.446
Drinking habit, yes/no; *n* (%)	21 (65.6)/11 (34.4)	211 (77.9)/60 (22.1)	0.122
Hypertension, yes/no; *n* (%)	15 (46.9)/17 (53.1)	78 (28.8)/193 (71.2)	0.032
Dyslipidemia, yes/no; *n* (%)	17 (53.1)/15 (46.9)	105 (38.7)/166 (61.3)	0.117
Hyperglycemia, yes/no; *n* (%)	10 (31.3)/22 (68.8)	40 (14.8)/231 (85.2)	0.006
Metabolic syndrome, yes/no; *n* (%)	6 (18.8)/26 (81.2)	36 (13.3)/235 (86.7)	0.327
Anti-hypertensive drugs, yes/no; *n* (%)	9 (28.1)/23 (71.9)	34 (12.5)/237 (87.5)	0.017
Anti-hyperlipidemic agents, yes/no; *n* (%)	7 (21.9)/25 (78.1)	18 (6.6)/253 (93.4)	0.003
Hypoglycemic drugs, yes/no; *n* (%)	2 (6.3)/30 (93.7)	5 (1.8)/266 (98.2)	0.117

The data are presented as the mean value (standard deviation) and the number of participants.

The abbreviations are the same as those in Table 1.

**Figure 2.**
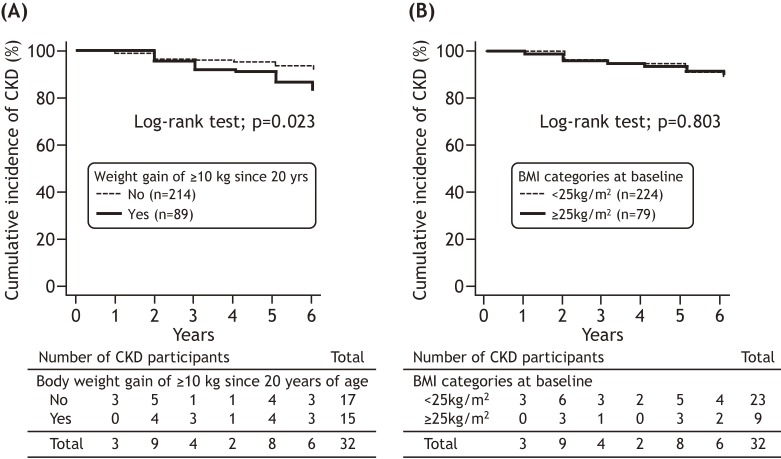
The cumulative incidence of CKD after 6 years follow-up period in participants with and without a body weight gain of ≥10 kg since 20 years of age (A) and current overweight (BMI at baseline ≥25 kg/m^2^, [B]). CKD, chronic kidney disease; BMI, body mass index.

**Figure 3.**
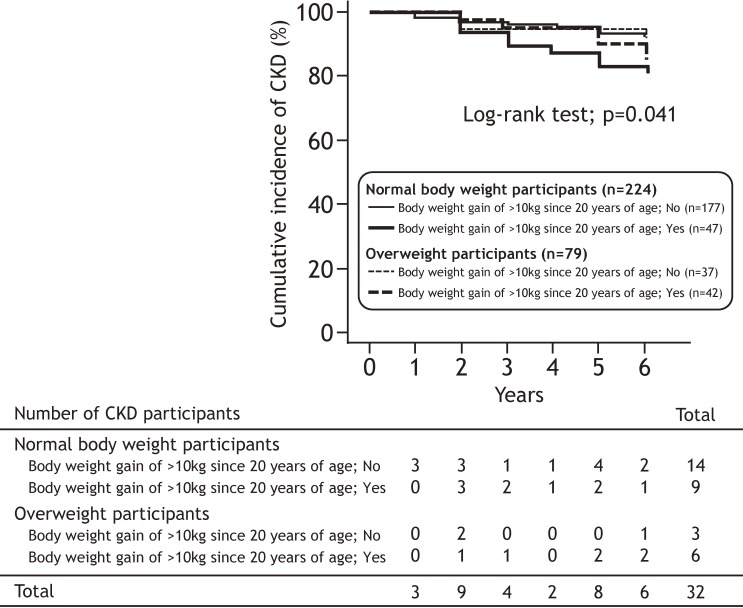
The cumulative incidence of CKD after 6 years follow-up period based on the combination of presence/absence of overweight participants with and without a body weight gain of ≥10 kg since 20 years of age. CKD, chronic kidney disease.

Figure 2 and Figure 3 show the cumulative incidence of CKD in participants with and without long-term body weight gain after maturity and overweight at baseline. When the participants were categorized according to long-term body weight gain after maturity, the cumulative incidence of CKD was significantly higher in participants with a body weight gain of ≥10 kg than in participants without body weight a body weight gain of ≥10 kg since 20 years of age (log-rank test: *P* = 0.023, Figure 2-A). However, there were no significant differences between current overweight and normal body weight (log-rank test: *P* = 0.803, Figure 2-B). The participants were also divided into four groups based on the combination of the presence/absence of current overweight with and without the long-term body weight gain after maturity. The cumulative incidence of CKD was significantly higher among the normal body weight participants with a body weight gain of ≥10 kg since 20 years of age (log-rank test: *P* = 0.041, Figure 3).

Table 3 indicates the relative risk of CKD progression during the follow-up period in the presence/absence of overweight participants with and without the long-term body weight gain after maturity. In a univariable analysis (model 1), the normal body weight participants with a body weight gain of ≥10 kg since 20 years of age was significantly associated with the incidence of CKD (hazard ratio [HR] 2.57; 95% confidence interval [CI], 1.11–5.93, *P* = 0.027). After adjusting for age, eGFR levels, the presence of metabolic syndrome, and smoking and drinking habits at baseline (model 2), the normal body weight participants with a body weight gain of ≥10 kg since 20 years of age also remained significantly associated with the incidence of CKD (HR 2.65; 95% CI, 1.12–6.23, *P* = 0.026). In another model, BMI, the presence of hypertension, dyslipidemia, and hyperglycemia, and smoking and drinking habits were entered as an adjusted factor instead of the presence of metabolic syndrome at baseline (model 3). In model 3, the normal body weight participants with a body weight gain of ≥10 kg since 20 years of age remained significantly associated with the incidence of CKD (HR 2.47; 95% CI, 1.02–6.01, *P* = 0.045).

**Table 3.** The influence of the combination of presence/absence of current body weight with and without the long-term body weight gain after maturity on the incidence of CKD

	Total	Developed CKD (*n*, %)	Univariable model		Multivariable model			
			(Model 1)		(Model 2)		(Model 3)	
		
			Hazard ratio (95% CI)	*P* value	Hazard ratio (95% CI)	*P* value	Hazard ratio (95% CI)	*P* value
Normal body weight participants (BMI at baseline <25 kg/m^2^)								
Body weight gain of ≥10 kg since 20 years of age								
No	177	14 (7.8)	1.00 (Ref.)	—	1.00 (Ref.)	—	1.00 (Ref.)	—
Yes	47	9 (19.1)	2.57 (1.11–5.93)	0.027	2.65 (1.12–6.23)	0.026	2.47 (1.02–6.01)	0.045
Overweight participants (BMI at baseline ≥25 kg/m^2^)								
Body weight gain of ≥10 kg since 20 years of age								
No	37	3 (8.1)	1.03 (0.30–3.60)	0.956	0.95 (0.26–3.42)	0.935	0.96 (0.23–4.14)	0.961
Yes	42	6 (15.0)	1.92 (0.74–5.01)	0.179	2.16 (0.78–6.02)	0.140	2.27 (0.63–8.11)	0.208

BMI, body mass index; CI, confidence interval; CKD, chronic kidney disease.

The data are presented as the hazard ratio (95% confidence interval [CI]).

In this analysis, the normal body weight and overweight with and without a body weight gain of ≥10 kg since 20 years of age at baseline was a dependent variable and the incidence of CKD was an independent variable. In the multivariate model, age, eGFR levels, presence of metabolic syndrome (metabolic syndrome diagnostic criteria of the Japanese Society for Internal Medicine^24^^,^^25^), and smoking and drinking habits at baseline were entered as adjusted factors (Model 2). In another model, BMI, the presence of hypertension (resting SBP ≥140 mm Hg and/or DBP ≥90 mm Hg and/or taking anti-hypertensive drugs), dyslipidemia (LDL-C ≥140 mg/dl and/or HDL-C <40 mg/dl and/or triglycerides ≥150 mg/dl and/or taking anti-hyperlipidemic agents), and hyperglycemia (fasting plasma glucose ≥110 mg/dl and/or HbA_1_c [NGSP values] ≥6.5% and/or taking hypoglycemic drugs) were entered as an adjusted factor instead of the presence of metabolic syndrome at baseline (Model 3).

The abbreviations are the same as those in Table 1.

## DISCUSSION

The major findings of the present study were that the relative risk for incidence of CKD was higher in the normal body weight participants with a body weight gain of >10 kg since 20 years of age than those without body weight gain after maturity. At present, the main risk factors for CKD are thought to be aging, obesity, hypertension, type 2 DM, and metabolic syndrome.^4^^–^^8^ Recently, long-term body weight gain after maturity has been associated with the progression of type 2 DM, metabolic syndrome, CVD, and cardiovascular mortality, independent of current body weight.^10^^–^^15^ Nanri et al^11^ investigated the relationship of body weight change since 20 years of age with the progression of type 2 DM, finding that long-term body weight gain after maturity increases the risk of type 2 DM, independent of current body weight. Wannamethee et al^13^ investigated the relationship between body weight change and the progression of CVD and cerebrovascular disease. Those authors showed that long-term body weight gain was associated with an about 30–70% increase in risk of CVD and cerebrovascular disease in participants with a basal BMI <27.5 kg/m^2^, but participants with basal BMI ≥30 kg/m^2^ demonstrated no such relationships. However, at present, the influence of long-term body weight gain after maturity on the progression of CKD has not been clarified. A previous prospective follow-up cohort study^28^ demonstrated that increases in body weight during the follow-up period were independently associated with increase in CKD risk, even if the BMIs remained in the normal range. Wakasugi et al^29^ cross-sectionally investigated the relationship of a body weight gain since 20 years of age with the prevalence of CKD. Those authors showed that long-term body weight gain after maturity was associated with the prevalence of CKD. According to our data, although a body weight gain of ≥10 kg since 20 years of age was associated with the incidence of CKD in participants with normal body weight, there were no significant relationships in overweight participants. In our previous study,^9^ we found that hypertension and hyperglycemia alone, as well as in combination, are risk factors for the incidence of CKD, independent of current overweight or metabolic syndrome. Based on these findings, long-term body weight gain after maturity in the normal body weight participants may be a sensitive factor for the progression of CKD, independent of current body weight, and may indirectly help prevent the progression of CVD or ESRD or the introduction of dialysis.

At present, long-term body weight gain after maturity is thought to induce the progression of CKD through pathways associated with insulin resistance and the accumulation of visceral fat, which can cause kidney injury, such as activations of the renin-angiotensin-aldosterone system, insulin/insulin-like growth factor-1 signaling pathways, oxidative stress, inflammation-related tissue damages, nephrosclerosis, and kidney sympathetic nervous system activity.^30^^–^^34^ Unfortunately, we were not able to clarify the causality of the relationship between long-term body weight gain after maturity and the progression of CKD, as the current research was performed within regular health checkups. However, it is well known that conventional risk factors for incidence of CVD, such as type 2 DM, hypertension, dyslipidemia, and their combination, are also associated with the deterioration of the kidney function.^9^^,^^35^^,^^36^ In participants with a body weight gain of ≥10 kg, the waist circumference, SBP and DBP, LDL-C, triglyceride, fasting plasma glucose, and HbA_1_c levels were higher and the HDL-C level lower compared with those without a body weight gain of ≥10 kg since 20 years of age. Given these present and previous findings, nephropathy, a leading cause of microvascular complications, may be caused by the long-term maintenance of insulin resistance and the accumulation of visceral fat and several cardiovascular risk factors since young adulthood, representing a possible mechanism underlying the association between long-term body weight gain after maturity and the progression of kidney dysfunction.

This study has several limitations. First, the study population was small, and was predominantly composed of middle-aged and older men with no health complications. It remains unclear whether the present results are applicable to women, participants with ESRD, or those with other health complications. Second, since the present study was performed within the participants’ regular health checkups, it was not possible to clarify the causality of the relationship between progression of CKD and long-term body weight gain after maturity. Furthermore, the current findings were not able to confirm the details of participants’ body weights at 20 years of age because long-term body weight gain after maturity was examined using a self-administered questionnaire. Finally, we calculated the eGFR using the Japanese GFR inference formula^20^ and evaluated proteinuria as an index of kidney function. To fully clarify the influence of long-term body weight gain after maturity on the progression of kidney dysfunction, other biomarkers, such as microalbuminuria and cystatin C, should be simultaneously assessed. However, we were not able to examine other biomarkers of kidney function in the present study.

However, despite these limitations, the current findings are the first to confirm the influence of the long-term body weight gain after maturity among normal body weight participants on the progression of CKD over a long follow-up period. The present results may support our hypothesis that long-term body weight gain after maturity leads to an increase in the incidence of type 2 DM, metabolic syndrome, CVD, and cardiovascular mortality, independent of current body weight.^10^^–^^15^ Given our results, we believe that administering the body weight from young adulthood is necessary in order to inhibit the progression of ESRD and CVD. Further investigations in a large number of participants are required to more precisely clarify the mechanisms, clinical implications, and relationships of the long-term body weight gain after maturity with the incidence of CKD.
